# National Diabetes Month — November 2019

**DOI:** 10.15585/mmwr.mm6843a1

**Published:** 2019-11-01

November is National Diabetes Month. In the United States, 30 million adults aged ≥18 years are living with diabetes and 84 million with prediabetes (*1*). Among persons aged ≥65 years, one in four is estimated to have diabetes, and one in two has prediabetes (*1*). Persons with prediabetes are at risk for developing type 2 diabetes, heart disease, and stroke (*2*). However, type 2 diabetes can be prevented or delayed through a structured lifestyle change program that promotes weight loss, healthy eating, and increased physical activity (*2*). A report on diabetes among Medicare beneficiaries is included in this issue of *MMWR* (*3*).

CDC plays a crucial role in preventing type 2 diabetes and diabetes complications. The National Diabetes Prevention Program (National DPP) (https://www.cdc.gov/diabetes/prevention/index.html) is a public-private partnership building a nationwide system to deliver an affordable, evidence-based lifestyle change program to prevent or delay type 2 diabetes. In 2018, the National DPP lifestyle change program became a covered service for Medicare beneficiaries with prediabetes. The first national prediabetes awareness campaign, DoIHavePrediabetes.org, done in collaboration with partners, encourages persons to learn their prediabetes risk. CDC also works to increase access to diabetes self-management education and support services to help persons with diabetes manage their daily diabetes care (https://www.cdc.gov/diabetes/dsmes-toolkit/index.html). More information is available at https://www.cdc.gov/diabetes.
